# Battling for Consumer's Positive Purchase Intention: A Comparative Study Between Two Psychological Techniques to Achieve Success and Sustainability for Digital Entrepreneurships

**DOI:** 10.3389/fpsyg.2021.665194

**Published:** 2021-05-14

**Authors:** Dandan Dong, Haider Ali Malik, Yaoping Liu, Elsayed Elsherbini Elashkar, Alaa Mohamd Shoukry, J. A. Khader

**Affiliations:** ^1^School of Journalism and Communication, Nanjing University, Nanjing, China; ^2^FAST School of Management, National University of Computer and Emerging Sciences, Islamabad, Pakistan; ^3^Department of Business Administration, Rajamangala University of Technology Krungthep, Bangkok, Thailand; ^4^Department of Business Administration, Mahidol University, Salaya, Thailand; ^5^Administrative Sciences Department, Community College, King Saud University, Riyadh, Saudi Arabia; ^6^Applied Statistics Department, Faculty of Commerce, Mansoura University, Mansoura, Egypt; ^7^Arriyadh Community College, King Saud University, Riyadh, Saudi Arabia; ^8^Department of Business Administration, KSA Workers University, Nsar, Egypt; ^9^College of Business Administration, King Saud University Muzahimiyah, Al-Muzahmiyya, Saudi Arabia

**Keywords:** purchase intentions, online purchase intention, perceived risk, social media, e-business, entrepreneurship, organizational sustainability

## Abstract

This research focuses on students' online purchase intentions in Pakistan toward different products available for sale on numerous e-business websites. This study's main objective is to determine which methodology is better to enhance customer online purchase intention. It also aims to discover how to improve perceived benefits and lower perceived risks associated with any available online product and entrepreneurship. AMOS 24 has been used to deal with the mediation in study design with bootstrap methodology. The study was conducted on 250 students from different educational institutes in Pakistan using a simple random sampling technique. A finding of this study suggests that both methods positively impact online purchase intention of consumers and sustainable digital economy. But social media advertisement is more effective through enhancing the perceived benefits of products. In contrast, product content factors are more effective at lowering the perceived risks associated with available online products.

## Introduction and Background

In recent decades, social media has been a valuable addition to our everyday lives (Schmid and Axhausen, 2019). As the technology has significantly flourished in the past few years, the effect of social networking sites have had a more significant and substantial impact on one's life than before (Ahmed et al., 2019). In the field of marketing, e-business and social media has drastically changed the competition of markets by being more efficient and significant (Heath, 2019). According to Chi, “social media marketing is a relationship between brands and consumers, which offers a personal channel and currency for user-centered networking and social interaction” (2011). Marketing through social media keeps consumers in the focus of the corporate world and entrepreneurships. It provides them with creative and innovative segments, allowing marketers to grab the consumers' attention and maximize efficient purchasing behavior (Vasić et al., 2019). The most significant and exciting feature is its speed and efficiency, as a potential customer is just one click away (Hossain, 2019) and it is only a few further clicks until a product is purchased. Therefore, it is not possible to grab the market without knowing its technological importance, and businesses must focus on attractive packages, policies, promotions, and offers to enhance purchasing behavior for a sustainable economy (Wai et al., 2019). Since the approach for interacting with customers has changed because of social media, businesses should learn how effectively they can use social media to improve their sales (Mangold and Faulds, 2009). The practical and smart use of social media benefits companies (Alam et al., 2020) struggling to get a competitive edge (Liu et al., 2008; Sarfraz et al., 2020a,b; Li et al., 2021).

It is essential to acknowledge that social media works as a platform where companies and customers interact directly for mutual benefits (Tandon, 2020). Hence, it is equally important to observe and interpret their customers' behavior to achieve the maximum benefits (Cao et al., 2018). According to an estimation, over 500 million people are using social media (Facebook) (Ostrow et al., 2019){#246}. It is impossible to read and address such a large number of individuals without social media help and entrepreneurships (Cao et al., 2018). Consumer behavior is most important in marketing, as (Huo et al., 2020) it helps and guides the marketers to plan their strategies and tactics more efficiently (Hair and Sarstedt, 2021). The consumer-socialization theory predicts that communication among consumers affects their affective (Harrigan et al., 2021), cognitive, and behavioral attitudes (Ward, 1974). Hence, if one happy customer exists, they will bring more customers because of their positive experience with a specific brand and will surely recommend it to friends or family. This cycle keeps on moving (Harrigan et al., 2021). When a particular brand focuses on their customers happiness, it creates a positive brand reputation among customers' friends (Lipsman et al., 2012). Therefore, when a product is marketed through social media, it has multiple chances of spreading considerably because of the consumer-socialization theory (Meire et al., 2019; Sarfraz et al., 2020c). So, it is vital to check how effectively companies use social media to attract and influence their targeted consumers apart from just marketing their products.

It is also imperative to remember that every person following social media is not the actual customer of that specific company or brand. So, it is equally crucial for a company to convert those random followers into loyal and happy customers. According to Lipsman et al. a fan's value can be analyzed in three ways: increasing the depth of loyalty and engagement among fans, generating incremental purchase behaviur, and leveraging fans' ability to influence friends. The major goal is to create a strong and impactful social media brand impression (Sheth, 2021). And this brand impression can convert the popularity of a company into actual financial value for the company. However, the basic need is to identify the key factors affecting the existing consumers, which will automatically help attract potential consumers to convert them into actual consumers through active marketing skills (Moorman et al., 2019).

## Objectives and Significance of the Study

The purpose of this study is (1) to comprehensively study the existing literature, which explains how companies and entrepreneurships through a digital economy use social media marketing tools and techniques (Vinerean et al., 2013). They use it to shape their e-business/marketing strategies in entrepreneurships to influence consumer behaviur regarding particular products (Moorman et al., 2019). (2) The chapter will introduce social media today and how it helps companies market their products to specific audiences worldwide. (3) The chapter will discuss different marketing techniques that are put into use through social media marketing and will explain how these techniques prove fruitful for companies and entrepreneurships and sustainable digital economy in terms of financial gains. Going a step further, the chapter will also discuss in detail the consumer behaviors and attitudes from the perspective of existing literature and will try to explain the factors which play a role in determining or changing the attitude of a consumer toward a product or a company he or she comes across on social media (Sarfraz, 2018; Dar et al., 2019, 2020).

Therefore, information from all relevant research streams will be incorporated in this chapter such that it paves the way for further research (Heinonen, 2011). The chapter will also serve as a foundation for primary research and help evaluate and understand the primary research results. Hence, the literature will be coupled with the results from real life to provide useful conclusions and recommendations about the subject under consideration and will try to answer the research questions in this context (Lopez and Castaño, 2019).

## Theoretical Background

**Figure 1:**
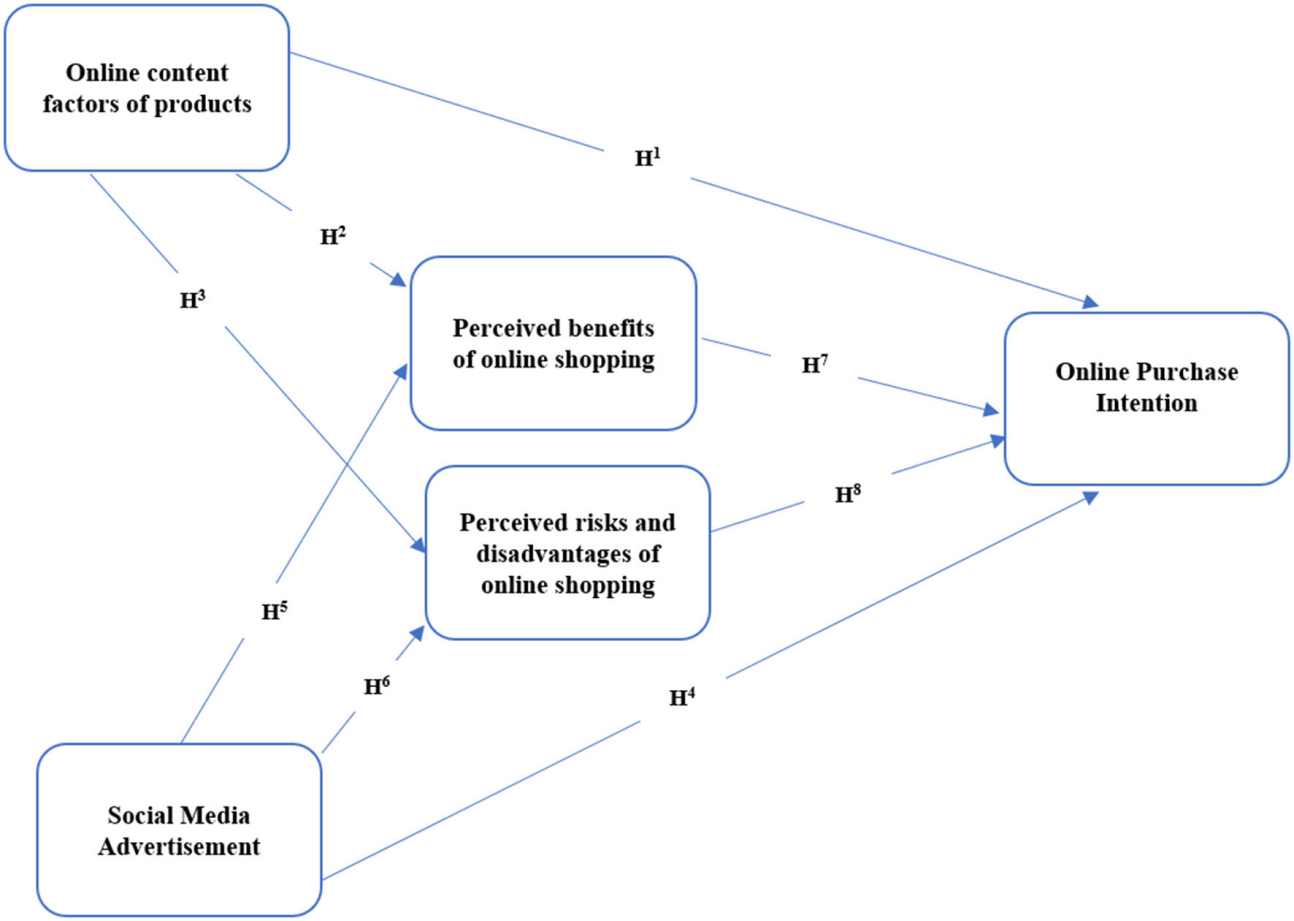
Theoretical framework of the study.

There are two mediating variables and two independent variables, given in Figure 1. These variables can influence the online digital purchase intention positively or negatively, which is a dependent variable. Moreover, it can enhance online shopping by giving or offering smooth and safe transactions in industrial organizations.

Consumer socialization theory forecasts that communication between consumers disturbs their affective, cognitive, and behavioral attitudes (Ward, 1974). Through socialization, consumers acquire consumption-related skills and attitudes toward the marketplace. The widely applied socialization framework delineates consumer knowledge processes and how people execute their parts as patrons in society (e.g., Moschis and Churchill, 1978; Churchill and Moschis, 1979; De Gregorio and Sung, 2010) and in the marketplace.

For a successful marketing relation with different variables, it is crucial to understand the importance of those variables or factors that directly or indirectly influence purchasing behavior. We explored the strong relationship between innovative design and social media advertisement and the perceived risks and benefits of online purchase intentions. We concluded that all of these critical factors are important for every marketer with strong cooperation required for a successful relationship. First, we observe and study the nature of relationship marketing and recommend how this theory should be abstracted. Then, we conclude that successful relationship marketing needs strong social media advertisement and unique product design, and discuss the perceived benefits and factors that reduce the amount of risk. Next, we present them as a prominent and significant mediating variable. After that, we test those major mediating variables using data to build a strong marketing relationship online. Finally, we make a comparison with our rival to show whether our model allows the most substantial connection of these mediating variables. It is found that online competition is increasing on a daily basis among the markets (Thorelli, 1986, p. 47).

## Literature Review

### Product Online Content Factors

Product design includes style, aesthetics, function, and overall product outlook as the basic components or elements that could be created for a specific product (Schivinski et al., 2021). A company, entrepreneurship, or brand mostly focuses on these features and elements to distinguish it from others (Naeem, 2021). Core product elements are the heart of any product, such as the engine system of an automobile or a computer processor, and they should not be compromised at all (Sultan et al., 2021) as it would be noticed if these elements differed from one another. These functions also allow the customer to distinguish between you and your competitor. Hence, unique product design's responsibility can directly stimulate the customer's buying behavior (Amen et al., 2020). Therefore, the design is a collective process through which differences could be created easily (Huo et al., 2020).

An advanced package of different design essentials has made it exciting and easy to distinguish products in looks and functions to an exclusively reserved position in the market. Regrettably, most products fail to attain distinction in this way and to present their product more uniquely than others. Understanding its importance will ultimately lead to product success (Li et al., 2020a). According to different research, the up-to-date view of the product began in the 1940's and 1950's. Other product designers were known as public figures and attained fame because of it (Loewy, 1950; Dreyfuss and Glimcher, 1955; Li et al., 2020b). Current designers must be efficient enough in all the essential or valuable fields, such as engineering, physical sciences, and social sciences (Molotch, 2005). A trio of impacts, from design, manufacturing, and marketing energies known as new product development and process of development. We started this study mainly to address the strategies, methods, goals, and tricks that product design teams can use to add value to customer attraction and satisfaction.

**H1:** Online content factors create a positive online purchase intention in the consumer's mind.

**H2:** Online content factors create a positive online purchase intention in consumers' minds when mediated by the perceived benefits of online shopping.

**H3:** Online content factors create a positive online purchase intention in consumers' minds when mediated by perceived risks and disadvantages of online shopping.

### Social Media Advertisement

This study aims to understand how brands and companies use social media advertisements to attract and influence consumer behaviurs toward their products (Voorveld et al., 2018). It is imperative to find and discuss how these attractions work to get maximum benefit (Alalwan, 2018). Understanding the foundations of social media marketing will help us evaluate how companies can use these tools to strengthen their brand image and thus (Alhabash et al., 2017) convert their followers into loyal buyers(Van-Tien Dao et al., 2014). It is also essential for a marketer to understand every social media feature before using it as a marketing or advertisement tool (Lee and Hong, 2016). According to Campbell et al. “social media is a lot more about how people are using the technology (Chu et al., 2013) and less about the technology itself. Because people are now creating and utilizing information instead of just storing it” (2011), social media can be defined as a tool or a complete package of different applications based on the technological base of web 2.0 which permits creating, innovating, or exchanging information through the internet (Chu et al., 2013). Furthermore, Web 2.0 could be explained more comprehensively as it is a totally new way where the information could be edited or created by the user anytime (Winter et al., 2021).

Purchase intention can be influenced because the contents of advertising and the message (Schivinski et al., 2021) through advertisement in social media are more eye catching and striking. Advertisements could be presented in numerous ways, like pictures, celebrity endorsement, or reviews of happy customers that could be uploaded (Naeem, 2021). The more powerful the message shared through visual advertisements, the more significant and positive a response could be collected from online consumers (Fotis et al., 2011). Wang et al. explains that the ads that appear very often leave a more significant impact on purchase intension. These ads do not have a specific message or maybe annoy the customer because of repetition (Sharma et al., 2021). Consumers may not resist a strong message or strong content that shares the product's innovating features and functions. The great increase in purchase intention will automatically lead to more sales (Hong and Kim, 2021).

**H4:** Social Media Advertisement creates a positive online purchase intention in the consumer's mind.

**H5:** Social Media Advertisement creates a positive online purchase intention in consumers' minds when mediated by the perceived benefits of online shopping.

**H6:** Social Media Advertisement creates a positive online purchase intention in consumers' minds when mediated by perceived risks and disadvantages of online shopping.

### Perceived Benefits of Online Shopping

Online shopping predicts many risks, and there is always room for benefits and attractions that ultimately manipulate consumers' minds toward online shopping (Katta and Patro, 2017a). The perceived or the important benefits are related to consumer's satisfaction and happiness with online shopping and the perception of a consumer or customer is that online shopping is supposed to be easy, convenient, trustworthy, time saving, less risky (Tzeng et al., 2021), and offer more variety compared to conventional shopping. The internet has changed our life completely; as everything is just one click away, in seconds we could comfortably be looking at the best products or services. As per the findings of Seiders et al. (2000), convenience offers different prospects in the online buying method: Find, acquire, get, and complete the transaction. Furthermore (González et al., 2021), convenience is an essential and vital motivation for online shopping (González et al., 2021).

Additionally, convenience is significant and positively related to buying behaviur, which encourages a buyer's willingness (Wang et al., 2007). Moreover, Wang et al. (2021) websites provide a massive variety for a specific brand to multiple different consumers at a time using single platforms because there is no problem involved with storing or displaying the stock. An additional type is a motivational factor that stimulates consumers to purchase online (Katta and Patro, 2017b). It is very convenient for consumers to stay at home and acquire any product they want (Al-Debei et al., 2015).

It saves time. Any busy individual can purchase their products in just a few clicks (Hebbar et al., 2020). Online shopping provides different coupons, sale offers, deals, promotions, and much more. So, it is more attractive in terms of benefits over risks.

**H7:** Perceived benefits of online shopping creates a positive online purchase intention in the consumer's mind.

### Perceived Risk and Disadvantages of Online Shopping

Perceived risk is well-defined by Dowling and Staelin (1994) as something that creates doubt in the consumer's mind to buy any specific goods or services. It has been demonstrated that perceived risk in consumer behavior is much more of an issue now than it has been in the past (Amirtha et al., 2021).

Product risk is the doubt a consumer has that the product will meet their expectations while making a purchase decision (Masri et al., 2021). Risk is experienced more in online shopping because the sense of physical experience and inspection is missing that cannot be passed on online (Tzeng et al., 2021). Traditional shopping provides better customer satisfaction as they can physically experience the product (Jain, 2021). That is why risk in traditional shopping is negligible as compared to advanced and speedy online shopping methods. It is the consumer's primary demand and concern (Lin et al., 2019). In shopping via technology, consumers have random or selective and very to-the-point information regarding a specific product. That is why they feel uncomfortable due to limited instructions (Lin et al., 2019).

Privacy risk is the most significant issue related to online shopping (Islam, 2021). Consumers are supposed and compelled to share very private details to make any transaction possible (Peng et al., 2019). Moreover, as online shopping grows in popularity, so too does the risk associated with it, causing people to not want to buy online. Especially in Pakistan, a there was a decrease of online buyers from 3 to 2.07%. Many people prefer cash on deliveries service because of that risk and do not want to share their bank details with the marketer or that website. According to Vasić et al. (2019), 8% of online users stopped buying online due to privacy risks and more than 50% do not even want to try online shopping because they feel it is very risky and they are afraid of any problematic situation (Nazione et al., 2021). Privacy risks automatically lead toward people not buying online. Moreover, it does not meaningfully attract the consumer to make a purchase decision. Consumers' concern and insecurity about their personal information or a specific product result in negative effects on intentions (Qalati et al., 2021).

The intention of a consumer to shop online could be improved by ensuring that their private data is the brand's priority. A few findings show that “privacy risk significantly reduces online shopping behavior” (Yildirim et al., 2021). Therefore, the connection between risk and shopping online is powerful, and it is essential to discuss this to obtain maximum benegit. In the most recent findings, purchase intention could be used to strengthen the relationship between risk and purchase behavior (Zhong et al., 2021). Furthermore, different studies predict that there is no need to focus on privacy risk because people are neglecting its consequences in online shopping behavior (Lazaroiu et al., 2020).

**H8:** Perceived risks and disadvantages of online shopping create negative online purchase intention in consumers' minds.

### Online Purchase Intention

Online purchase intention is the choice of an individual to purchase anything through the internet (Jain, 2021). While making a purchasing decision, the purpose could be affected by many factors that play vital roles like trust, time-saving, and convenience. If a lack of consumer purchase intention exists, it might cause significant problems because that specific person might influence others' behavior toward online shopping who are loyal or happy customers (Ma et al., 2021). Additionally, intentions are a collection of thoughts on whether an individual is willing to purchase or their specific buying behaviors (Jain, 2021).

Meanwhile, attractions through benefits and risks are the boosters of actual consumers' behavior. Most of the research describes a healthy and positive relationship among online shopping and purchase intentions (Chen et al., 2021; Ham and Chung, 2021) Many of the researchers found that it will help if our focus was on consumer purchase intention because it works well in online shopping to maintain a sustainable digital economy (Bhatti et al., 2018). There is an emerging trend to prioritize the latest trends in behaviur for the future because the future is all about online transactions (Chen and Zimitat, 2006; Bhatti et al., 2018).

#### Research Methodology

A hypothesis study has been used for this research to explain the nature of the relationship between a number of variables. Students from different educational institutes were drawn from the admission offices of their respective educational institutes. The reason for selecting students from universities is that they are heavy users of social media. They also have the highest probability of buying products online with the ability to spend money in hand.

This study used a correlational type of investigation because it needs to check the variables' relationship through hypotheses. Research has been conducted in a natural environment. That is why it will be considered a non-contrived study setting. This study has minimal researcher interference toward respondents regarding the filling out of questionnaires. In this study, the data is collected from students; that is why this study's unit of analysis is individual. The researcher has implemented a cross-sectional study method for this study. It involves the study of a whole population, or a representative subset, at one specific point in time.

#### Empirical Settings and Data Collection

The data was obtained with respondent's consent. These studies have been conducted on students from different universities and educational institutes of the Punjab province; 500 questionnaires were distributed among them, and almost 270 questionnaires were returned, maintaining a response rate of 55%. A reliable and valid questionnaire has been used for this study. As 20 questionnaires were returned with incomplete information, the analysis was done with 250 complete responses. Respondents' demographic profile is given in Table 1.

**Table 1:** Respondents' demographic profile.

**Category**	**Subdivision**	**Frequency**	**Percentage**
**Demographic profile of the respondents**			
Marital status	Married	150	60
	Un-married	100	40
Age	Below 25 years	50	2
	25–30	85	34
	31–35	65	26
	36–40	40	16
	40 and above	10	4
Education	Intermediate	110	44
	Bachelors	128	51
	Masters	12	5
	M.Phil	0	0
	Phd	0	0
Internet usage frequency	Once in a day	202	80.5
	After 3 days	17	6.8
	After 1 week	6	2.4
	After 2 weeks	2	8
	After 1 month	24	9.6

The respondents were students from a variety of colleges and universities and were selected randomly using a simple random sampling technique. Respondents had to have experience using social media or online purchasing websites so that they could answer the questionnaire with more information and awareness.

## Measure and Methods

### Instrument

For measuring online content factors of products, perceived benefits of online shopping, and perceived risks and disadvantages of online shopping, we will use the scale developed by Adnan (2014). We will use the scale developed by Logan et al. (2012) and online purchase intention. We will use a scale developed by Duffett (2015). The instruments were rated and measured on a 5-point Likert scale with higher numerical values showing greater satisfaction.

### Confirmatory Factor Analysis

It is necessary to conduct the confirmatory factor analysis for accurate and precise results for all variables. For this study, it was decided to conduct a pooled CFA analysis, which is given in Table 2. It runs all the latent variables at the same time to achieve the required model fitness. The pooled CFA method is a lot easier and better than the individual CFA since it runs all the latent variables simultaneously, which is time-saving (detail given in Table 3) (Afthanorhan et al., 2014; Chong et al., 2014).

**Table 2:** Pooled CFA model fitness tests.

**Name of category**	**Name of index**	**Index full name**	**Value in analysis**	**Acceptable value**	**References**
**Pooled CFA model fitness tests**					
Absolute fit	RMSEA	Root mean square of error approximation	0.049	<0.80	Browne and Cudeck, 1993
Incremental fit	CFI	Comparative fit index	0.938	>0.90	Bentler, 1990
Parsimonious fit	Chisq/df	Chi Square/Degrees of freedom	1.590	<3	Hu and Bentler, 1999

**Table 3:** Pooled confirmatory factor analysis (Independent, mediating, and dependent variable).

**Scale**	**Items**	**Factor loadings**	**Scale reliability**
**Pooled confirmatory factor analysis (independent, mediating, and dependent variable)**			
Online content factors of products	I buy from online stores only if they are visually appealing and have a well-organized appearance.	0.739	0.719
	I buy from online stores only if the navigation flow is user friendly.	0.740	
	I buy from online stores only if the site content is easy for me to understand and the information provided is relevant.	0.656	
	I buy from online stores only if they have an easy and error free ordering and transaction procedure.	0.742	
Social media advertisement	Social media advertising is a good source of product information and supplies relevant product information.	0.770	0.777
	Social media advertising provides timely information.	0.993	
	Social media advertising is a good source of up-to-date product information.	0.558	
	Social media advertising is a convenient source of product information.	0.856	
	Social media advertising supplies complete product information.	0.708	
Perceived benefits of online shopping	I shop online as I can shop whenever I want to (24/7 availability).	0.707	0.703
	I shop online as I get detailed product information online.	0.739	
	I shop online because I get a broader selection of products and better deals available.	0.640	
	Online shopping gives the facility of easy price comparison (Hence, price advantage).	0.701	
	I shop online as I get user/expert reviews on the product.	0.776	
	I use online shopping for buying products which are otherwise not easily available in the nearby market or are unique/new	0.656	
	I shop online as there are more payment options available.		
Perceived risks and disadvantages of online shopping	I hesitate to shop online as there is a high risk of receiving malfunctioning merchandise.	0.742	0.760
	It is hard to judge the quality of the merchandise over the internet.	0.802	
	I feel that there will be difficulty in settling disputes when I shop online (e.g., while exchanging products).	0.816	
	I might not receive the product ordered online.	0.605	
	I do not like being charged for shipping when I shop online.	0.775	
	Getting good after sale service is time taking and difficult for online purchases.	0.825	
Online Purchase Intention	I will buy products that are advertised on social media.	0.825	0.709
	I desire to buy products that are promoted on advertisements on social media.	0.763	
	I am likely to buy products that are promoted on social media.	0.543	
	I plan to purchase products that are promoted on social media.	0.705	

The model fit indices show an acceptable fit between the data and the proposed measurement model. The values of the Comparative Fit Index (CFI = 0.938), Root Mean Error of Approximation (RMSEA = 0.049), and Chi-square to Degree of Freedom Ratio (x 2/df = 1.590) all meet the cutoff criteria, so the values of the fitness indices meet the excellent standards for model fitness (Lomax and Schumacker, 2004; Hoe, 2008; Anderson et al., 2010).

After running the pooled CFA, it is also necessary to check and verify each item's reliability for further research. CFA of this study's data was used to measure reliability, convergent validity, and discriminant validity. The reliability of the measurement scales was measured with composite reliability, which is preferred to report a scale's reliability (Netemeyer et al., 2003).

Discriminant validity is used to confirm that the measurement scales are distinct from other measures used in the study. Discriminant validity was measured using the HTMT analysis in which the cut-off criteria for strict discriminant validity is 0.850 and for liberal discriminant validity is 0.900 (details given in Table 4) (Henseler et al., 2015). Therefore, it is established that all the measurement scales used in the study differ from each other, so the data used in our study fulfils the requirements of convergent and discriminant validity and is suitable for further analysis.

**Table 4:** HTMT analysis to measure discriminant validity.

	**Content factor**	**Social media advertisement**	**Perceived benefits**	**Perceived risks**	**Online purchase intention**
**HTMT analysis**					
Content factor					
Social media advertisement	0.275				
Perceived benefits	0.272	0.167			
Perceived risks	0.107	0.095	0.050		
Online purchase intention	0.320	0.070	0.055	0.578	

### Structural Equation Modeling

Structural equation modeling (SEM) was used in the structural model to test the hypotheses, using AMOS 24 (detail given in Table 5). As the proposed model contains mediation, the SEM technique was used to analyze all the paths simultaneously (Iacobucci et al., 2007; Hoe, 2008; Alavifar et al., 2012). The model fit indices for the structural model meet the acceptance criteria.

**Table 5:** Structural equation modeling analysis.

**Name of category**	**Name of index**	**Index full name**	**Value in analysis**	**Acceptable value**	**References**
**SEM, model fitness tests**					
Absolute fit	RMSEA	Root mean square of error approximation	0.067	<0.80	Browne and Cudeck, 1993
Incremental fit	CFI	Comparative fit index	0.915	>0.90	Bentler, 1990
Parsimonious fit	Chisq/df	Chi square/degrees of freedom	1.214	<3	Hu and Bentler, 1999

### Hypothesis Testing

The results of the structural model are shown in Table 6. The SEM statistics show that **H1 (Content Factors**→**Purchase Intention)** and **H4 (Social Media Advertisement → Purchase Intention)** are rejected on the grounds of significance level, as the SEM results show that the *P*-values of these hypotheses are not significant. These results suggest that these variables do not have a direct significant positive impact on employee loyalty. While **H7 (Perceived benefits → Purchase Intention)** and **H8 (Perceived Risks → Purchase Intention)** are accepted on the grounds of significance level, as the SEM results show that the *P*-values of these hypotheses are significant. These results suggest that these variables have a direct significant positive impact on employee loyalty. Moreover, the results also indicate that high perceived benefits could lead to positive purchase intention, directly proportional to independent and dependent variables. In contrast, the higher perceived risk could lead toward negative purchase intention and vice versa.

**Table 6:** Direct findings of the SEM.

**Hypothesis**	**Causal path**	**Lower bound**	**Upper bound**	***P*-value**	**Standardized estimated**
**Results of structural model: direct effects**					
H1	Content factors → Purchase intention	−0.162	0.093	0.790	−0.032
H4	Social media advertisement → Purchase intention	−0.183	0.026	0.306	−0.080
H7	Perceived benefits → Online Purchase intention	0.096	0.378	0.005	0.335
H8	Perceived risks → Online Purchase intention	0.219	0.464	0.003	0.430

These results shown in Table 7 display the complete picture of this research study. The study showed that **H2** (**Content Factors → Perceived Benefits → Online Purchase Intention**, **β = 0.20**, ***P* = 0.005**) is positively significant and suggests that when websites impressively use the product content factor then it is effective in enhancing the product's perceived benefits in the eyes of its target customer, hence leads toward positive online purchase intention behavior.

**Table 7:** Indirect findings of the SEM.

**Hypothesis**	**Causal path**	**Lower bound**	**Upper bound**	***P*-value**	**Standardized estimated**
**Results of structural model: indirect effects**					
H2	Content factors → Perceived Benefits → Purchase intention	0.060	0.174	0.005	0.20
H5	Social media advertisement → Perceived benefits → Purchase intention	0.027	0.140	0.045	0.55
H3	Content factors → Perceived Risks → Purchase Intention	0.052	0.153	0.026	0.35
H6	Social media advertisement → Perceived risks → Purchase intention	0.019	0.098	0.009	0.25

The study showed that **H3 (Social Media Advertisement → Perceived Risks → Online Purchase Intention**, **β = 0.55**, ***P* = 0.045)** is also positively significant and suggests that organizations that use social media advertising to promote their products online create a positive impact in their targeted customer's online purchase intentions.

This hypothesis showed that **H5** (**Content Factors → Perceived Risks Online → Purchase Intention**, **β = 0.35**, ***P* = 0.026**) is positively significant and suggests that content factors of the available online create a positive online purchase intention when perceived risks mediate it. Hence, it could be deducted that content factors help lower the perceived risks in buyers' minds and enhance their online purchase intention toward that specific product.

This specific hypothesis showed that **H6 (Social Media Advertisement → Perceived Risks → Purchase Intention**, **β = 0.25**, ***P* = 0.009**) is also positively significant and suggests that organizations' investment on social media advertisement is useful and creates a positive online purchase intention in its target customers.

## Discussion

This study's primary purpose was to encounter all the variables that may increase or decrease intention toward a valuable consumer's purchase behavior. There are multiple significant and positive relationships or dimensions that may influence an individual's shopping online.

Innovative and creative design, ads through social media, and benefits and risks related to online purchase intentions directly affect consumers' buying behavior. So, the marketers have a significant gap to capture the market entirely and create a competitive edge.

In this research, we found that married people between 25 and 30 are more inclined to shop online. So, existing marketers can mold their advertisements according to this age group's interest, and they will automatically influence their social circle and community.

More benefits like giving a cash on delivery option or providing them with a trial option makes consumers feel the experience is more convenient and are happier about that product. It will create a significant difference among marketers.

On the other hand, the risk could be controlled more efficiently by providing the customer with ease and choices. The shared findings show that the relationship between benefits and risks is not only important in marketing relationship, but also that there are many more factors which are equally important and demand investigation in entrepreneurship (Becker, 1960; Achrol, 1991; Dwyer et al., 2001). They are also key mediating variables in these relationships. We found that the relationship among these variables is significantly and positively related with the desired outcomes.

Moreover, more variables could be added to get different opinions on where to work and how to work, especially for online shopping. A specific gender could be chosen to get other markets, but the variables discussed in this research give a clear direction for the existing and new markets.

## Conclusion

This study showed that both techniques are helpful in enhancing the online purchase intention of target customers while mediated by a product's perceived risks and benefits. But for specific actions, social media advertisement is more useful in enhancing the perceived benefits of those products available for sale online (Michaelidou et al., 2011; Kim and Ko, 2012). At the same time, the content factors or product listing is more helpful in lowering the perceived risks associated with any available product online. These conclusions are also backed up by other studies conducted (Hong et al., 2004; Schmutz et al., 2010; Boateng and Okoe, 2015). Moreover, the impact of control variables still needed to be discussed in this research work. The specific variables which are selected for this research could perform indifferently in different situations. The results of this study could help organizations promote their product and services so that they could minimize their promotional costing of that product, lower the perceived risks associated with their product, and elevate the perceived benefits effectively.

Finally, this study's results could also vary from time to time due to demographics and geographic changes. That is why it is strongly recommended to apply this research framework in other situations or even the same problem again to verify and generalize the said results.

Despite these collaborative and managerial implications, this study has numerous limitations that provide salient future research directions. First, the websites included were not categorized according to region, so the respondent data was not associated with norms and cultural background. So, there is much more work required regarding this aspect. In addition, there are different marketing techniques to enhance purchase intention.

## Data Availability Statement

The original contributions presented in the study are included in the article/supplementary material, further inquiries can be directed to the Corresponding author/s.

## Author Contributions

DD: writing main draft and analysis. HA: revised the draft, data collection, and improved the article. YL: lead this study, final draft, and analysis. EE: layout, framework, and analysis elucidation. AM and JK: data, proof read, and language editing. All authors contributed to the article and approved the submitted version.

## Conflict of Interest

The authors declare that the research was conducted in the absence of any commercial or financial relationships that could be construed as a potential conflict of interest.
